# Cartilage Conduction Sounds in Cases of Wearing Different Transducers on a Head and Torso Simulator with a Manipulated Ear Pinna Simulator

**DOI:** 10.3390/audiolres13060078

**Published:** 2023-11-09

**Authors:** Ryota Shimokura, Tadashi Nishimura, Hiroshi Hosoi

**Affiliations:** 1 Department of Systems Science, Graduate School of Engineering Science, Osaka University, D436, 1-3 Machikaneyama, Toyonaka 560-8531, Osaka, Japan; 2 Otolaryngology—Head & Neck Surgery, Nara Medical University, 840 Shijo-cho, Kashihara 634-8522, Nara, Japan; t-nishim@naramed-u.ac.jp; 3 President and Medicine-Based Town Institute, Nara Medical University, 840 Shijo-cho, Kashihara 634-8522, Nara, Japan; hosoi@naramed-u.ac.jp

**Keywords:** cartilage conduction, pinna simulator, head and torso simulator, sound pressure level

## Abstract

Cartilage conduction is known widely as a third hearing transmission mechanism after the air and bone conduction methods, and transducers dedicated to the production of cartilage conduction sounds have been developed by several Japanese companies. To estimate the acoustic performance of the five cartilage conduction transducers selected for this study, both airborne sounds and cartilage conduction sounds were measured. Airborne sounds can be measured using a commercial condenser microphone; however, cartilage conduction sounds are impossible to measure using a conventional head and torso simulator (HATS), because the standard-issue ear pinna simulator cannot reproduce cartilage conduction sounds with the same spectral characteristics as the corresponding sounds measured in humans. Therefore, this study replaced the standard-issue simulator with a developed pinna simulator that can produce similar spectral characteristics to those of humans. The HATS manipulated in this manner realized results demonstrating that transducers that fitted the entrance to the external auditory canal more densely could produce greater cartilage conduction sounds. Among the five transducers under test, the ring-shaped device, which was not much larger than the entrance to the canal, satisfied the spectral requirements.

## 1. Introduction

**Figure 1:**
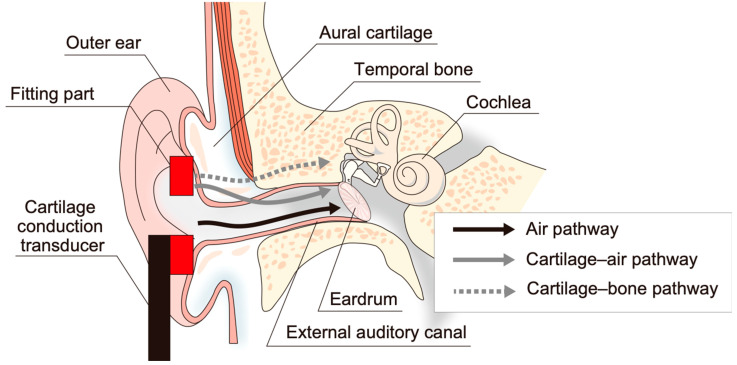
Possible transmission pathways when a transducer is placed on the aural cartilage.

Cartilage conduction offers a sound transmission pathway into the cochlea, in addition to the air and bone conduction routes [1,2,3]. The human aural pinna and the exterior half of the external auditory canal are composed of aural cartilage, in which amplified sound propagates when a transducer touches the aural cartilage. The transmission pathways by which the sound reaches the cochlea can be assumed in the following three cases to be as shown in Figure 1 [4,5]. The first pathway is that where the airborne sound from the transducer arrives at the ear drum directly through the external auditory canal (air pathway). In this case, the aural cartilage does not intervene in the hearing process. The second pathway is that where the oscillated cartilage generates the sound in the canal, and this sound then propagates through the eardrum and the middle ear (cartilage–air pathway). The third pathway is the case where the vibration of the cartilage is transmitted into the skull bone (cartilage–bone pathway). Our acoustic measurements and psycho-acoustic experiments have proved previously that the cartilage–air pathway contributes in a dominant manner to the hearing of users without any disorder of the outer ear [4,5,6]. Yazama et al. (2023) confirmed transmitted vibrations at ear ossicles (i.e., middle ear) by using a non-contact laser Doppler vibrometer when a transducer was stimulated at the ear tragus of participants under cochlea implant surgery [7]. Because sounds in the air and cartilage–bone pathways are classified as only airborne and bone-borne sounds, respectively, the unclassified sound transmitted through the cartilage–air pathway is referred to as the third pathway [3]. Besides cartilage conduction, the third form of hearing has been introduced in various research (e.g., non-osseous bone conduction [8], body conduction [9], ankle audiometry [10,11], or distantly presented bone conduction perception [12]). However, in this study, the used transducers actively stimulated the aural cartilage, so it is reasonable to define it as cartilage conduction.

**Figure 2:**
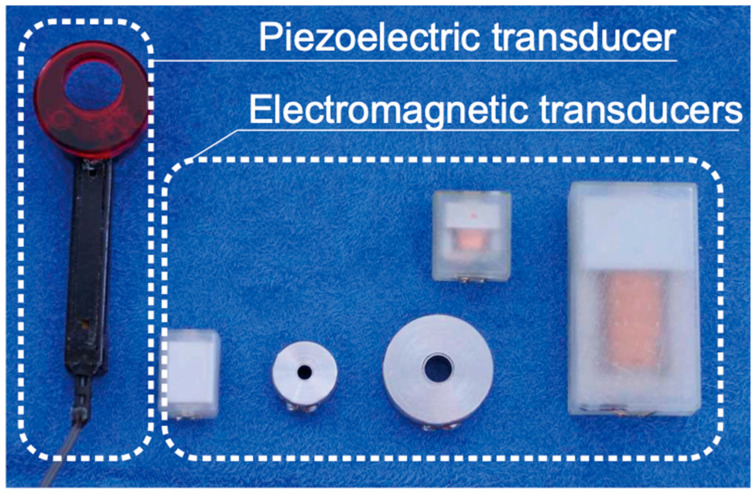
Piezoelectric transducer and electromagnetic transducers developed in our group.

**Figure 3:**
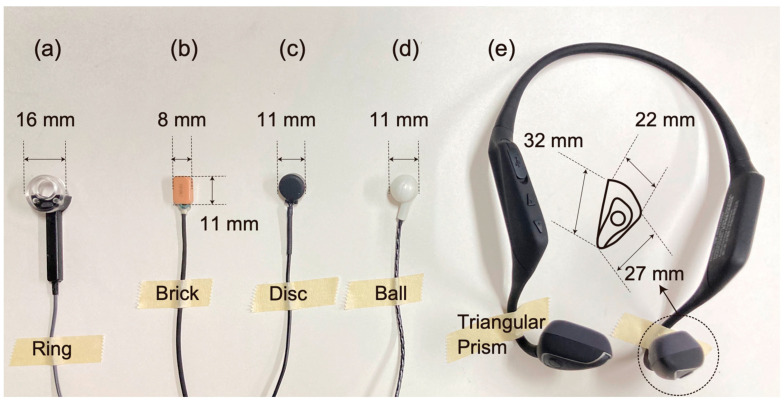
Five cartilage conduction transducers used in this study. (**a**–**e**) Photos and dimensions for transducers A to E, respectively.

The main performance requirement for a cartilage conduction transducer is to transmit vibrations to the aural cartilage effectively. As shown in Figure 2, the first transducer was designed in a ring shape to gain a contact surface with the entrance to the auditory canal [13]. The ring shape shown can produce sound without occluding the external auditory canal; however, the standing wave formed in the canal ensures that the sound leakage is minimized [14]. A piezoelectric bimorph covered in elastic material is built in the shaft part and an acrylic ring (fitting part) is connected to the bimorph. In most papers at the beginning of our cartilage conduction research, the first transducer type was used (e.g., [4,5,6,13,14]). In this study, we compared the output performances of successive cartilage conduction transducers when cartilage conduction was induced. The piezoelectric transducer is one of the target transducers assessed in this study (Figure 3a).

To minimize the transducer size, we developed electromagnetic transducers in anticipation of their use in commercial release hearing aids (Figure 2). Finally, the Japanese hearing aid manufacturer RION Co., Ltd. (Kokubunji, Japan) developed their first electromagnetic transducer embedded in a cartilage conduction hearing aid, as shown in Figure 3b [15]. The cartilage conduction hearing aid was developed to support conductive hearing losses (e.g., atresia of the external auditory canal and the otorrhea). Therefore, the transducers that are available in the market are covered with custom-made acrylic ear plugs, because they do not need to maintain ventilation with respect to the external auditory canal. Although patients with the conductive hearing loss are generally counseled to use bone conduction hearing aids, cartilage conduction hearing aids realize similar hearing thresholds after fitting the bone conduction and bone-anchored hearing aids [16,17]. In this study, the electromagnetic transducer was used without a cover to retain the gap with respect to the entrance to the auditory canal (Figure 3b).

After the release of the cartilage conduction hearing aid above, a company specializing in the manufacture of cartilage conduction transducers, CCH Sound Co., Ltd. (Kyoto, Japan), was established [18]. This company has developed two types of mass-produced transducer (the CCH sound disk and the CCH sound ball), which were optimized to induce cartilage conduction while also maintaining the existing market prices. Electromagnetic drivers were applied in these transducers. The CCH sound disk was not designed to fit on the canal entrance because it is a mounted component, while the CCT sound ball is designed to be fixed on the canal entrance, as shown in Figure 3c and Figure 3d, respectively. The CCH sound disk and the CCH sound ball were used in their factory shipped states in this study.

Based on the existing patents and through consultation with CCH Sound, the audio equipment maker Audio Technica Co., Ltd. (Machida, Japan) developed the world’s first earphone specifically for cartilage conduction hearing [19]. The two transducers are connected via a flexible wire arm and placed on the ear tragi to hold the head (Figure 3e). As a result, these transducers do not occlude the external auditory canals and it is recommended that the user does something such as listen to music in the background. The transducer can be connected to a player through Bluetooth. Because the stimulation at the temple is unsuitable for transmitting sound via bone conduction [20], the cartilage conduction sound may support the primary contribution of hearing by this device.

The purpose of this study was to compare the cartilage conduction sounds produced by the five transducers (Figure 3) that were specifically developed to generate them. Before the measurement of the cartilage conduction sounds, the outputs for airborne sounds were measured using a 1/2-in condenser microphone. The cartilage conduction sounds were simulated using a head and torso simulator (HATS), in which the ear pinna simulator had been replaced with the specially designed simulator to realize the cartilage conduction sounds [21]. The new simulator was used because the default pinna simulator used in the HATS is made from silicone rubber and is too soft to reproduce a spectral shape that is the same as that of the measured cartilage conduction sounds in humans [22]. The limitation of such an artificial head has also been reported in research on hearing protection [23,24]. As described above, cartilage conduction is not classified as airborne sound because the sound source is simply part of the body (i.e., the aural cartilage). Therefore, the HATS that is commonly used for the calibration of air conduction hearing aids [25] is useless for the evaluation of cartilage conduction sounds. In our previous study, we found that the hardness of the pinna simulator should match that of the actual aural cartilage and skin (durometer hardness: A10 to A20) to simulate cartilage conduction sounds [26], although the hardness of the pinna shows a large deviation according to the measurement equipment and individuals [27,28,29]. Because the pinna simulator of the HATS is removable from the body, we fabricated a mold that enabled us to form new pinna simulators with three different hardnesses (A10) [21]. Although the modified HATS was specifically constructed for cartilage transducers, there remain some errors in terms of the spectral representation, which are referred to in the discussion section. This study concentrates solely on comparing the different performances among cartilage conduction transducers. In previous studies related to cartilage conduction, a few types of transducers were used [4,5,6,7,14]. The main novelty of this study is to clarify the optimal shape and configuration for stimulating the aural cartilage.

## 2. Method

### 2.1. General Methods

The input signal to the transducer was a pure-tone train with frequencies ranging from 125 Hz to 16 kHz in 1/12 octave steps. The tones were 1 s in duration and each tone was followed by a 0.5 s long silent interval. The input levels were varied according to the transducer type. For the piezoelectric transducer (termed Transducer A in Figure 3a), the input levels were 2, 1, and 0.5 V; however, for the electromagnetic transducers (Transducers B, C, D, and E in Figure 3b, Figure 3c, Figure 3d, and Figure 3e, respectively), the input levels were 0.2, 0.1, and 0.05 V to adjust the differences for efficient amplification. The sound pressure levels (SPLs) were determined based on the spectral peaks at the corresponding pure-tone frequencies. In this study, the differences in the input levels were conveniently termed the high, middle, and low inputs in descending order.

The pure tones, which were recorded using a condenser microphone and the HATS (see Section 2.3 and Section 2.4 for further details), were adjusted using a conditional amplifier (NEXUS; Brüel & Kjær, Naerum, Denmark). Both the output and input data were digitized at a sampling rate of 44.1 kHz and with 16-bit resolution via an analog-to-digital/digital-to-analog (AD-DA) converter (Fireface UCX, RME, Haimhausen, Germany); the resulting data were then controlled using a PC (MacBookPro; Apple, Cupertino, CA, USA). The sound recordings were made in a soundproof chamber with background noise of less than 30 dB.

### 2.2. Cartilage Conduction Transducers

The five specialized transducers for cartilage conduction (Transducers A to E) are shown in Figure 3. Transducers A to D have line connections and transducer E is available via a Bluetooth connection. Although transducer E has two drivers for the left and right sides, only the right side was used during the measurements. Although transducer E has several digital signal processing (DSP) options, it was reset to its factory settings. Since all the transducers maintain the ventilation of the external auditory canal, the occlusion effect, which is known for increasing sound pressure below 1.2 kHz, could be minimized [30,31,32,33].

### 2.3. Measurement of Airborne Sound

**Figure 4:**
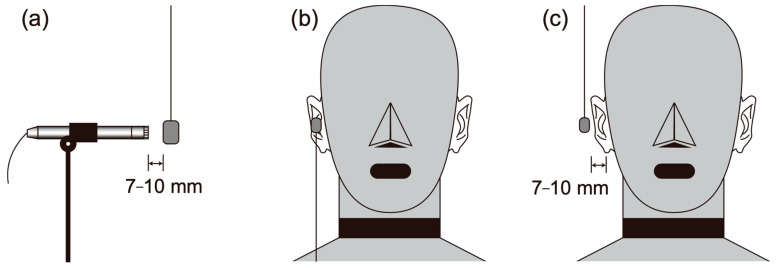
Measurement conditions for (**a**) airborne sound and cartilage conduction sound under (**b**) the touching condition and (**c**) the non-touching condition.

To evaluate the simple acoustic output, the signals were measured using a 1/2-in condenser microphone (4191, Brüel & Kjær, Naerum, Denmark), which was separated from the transducers by a distance of 7 to 10 mm (Figure 4a). The transducers were hung in order to face the vibrating surfaces toward the diaphragm of the microphone.

### 2.4. Measurement of Cartilage Conduction Sound

**Figure 5:**
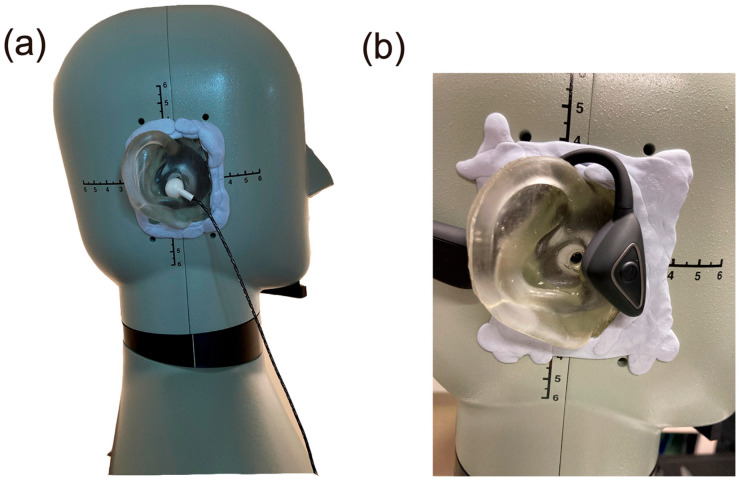
Head and torso simulator (HATS) with the hardness-adjusted pinna simulator with (**a**) transducer D and (**b**) transducer E.

To evaluate the cartilage conduction sounds, the signals were measured using the right ear of a HATS (4128, Brüel & Kjær, Naerum, Denmark). As shown in Figure 5, the existing pinna simulator of the right ear was replaced with our pinna simulator, the hardness of which was adjusted to reproduce the cartilage conduction sound more correctly [21]. The spatial gap between the pinna simulator and the HATS body was filled using rubber cement (Blu Tack, Bostik Australia Pty. Ltd., Thomastown, Australia) to prevent sound leakage.

To estimate the cartilage conduction gains, we performed the measurements under two conditions. The first condition involved placing the transducer in contact with the pinna simulator (the touching condition shown in Figure 4b); in the second condition, the transducer was placed in essentially the same position, but without touching the aural cartilage (the non-touching condition shown in Figure 4c). Because the transducer generated a collateral airborne signal (the air pathway shown in Figure 1), the difference between the SPLs achieved under these two conditions allowed us to specify the amount of the signal to be transmitted through the cartilage–air pathway alone (cartilage–air pathway shown in Figure 1) [4]. Transducers A to D were placed on the entrance to the canal, and transducer E was placed on the ear tragus (Figure 5b) in the touching condition. In the non-touching condition, transducers A to D were hung and transducer E was disconnected from the ear tragus by inserting a small piece of rubber cement between the flexible wire arm and the HATS body.

## 3. Results

**Figure 6:**
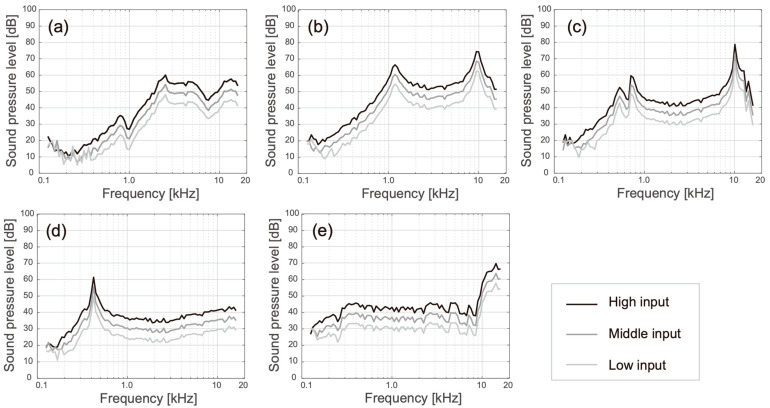
Sound pressure level (SPL) of airborne sound as a function of frequency. (**a**–**e**) Represent the results for transducers A to E, respectively.

Figure 6 shows the SPLs for the airborne sounds radiated from the five transducers. Because the input levels of the piezoelectric and electromagnetic transducers differed and transducer E received unknown amplification from the DSP, the values for the five transducers were not comparable. However, their specific spectral gains could be found. The SPL of transducer A showed a low-pass-like filter characteristic that decayed below approximately 2.5 kHz, but remained relatively flat above that frequency (Figure 6a). The spectral shapes of transducers D and E were the flattest among the five transducers and they showed one resonance peak each in the low- (420 Hz) and high (14 kHz)-frequency ranges, respectively (Figure 6d,e). Transducers B and C both had two resonance peaks that made their spectral shapes look like bandpass filter characteristics (Figure 6b,c).

**Figure 7:**
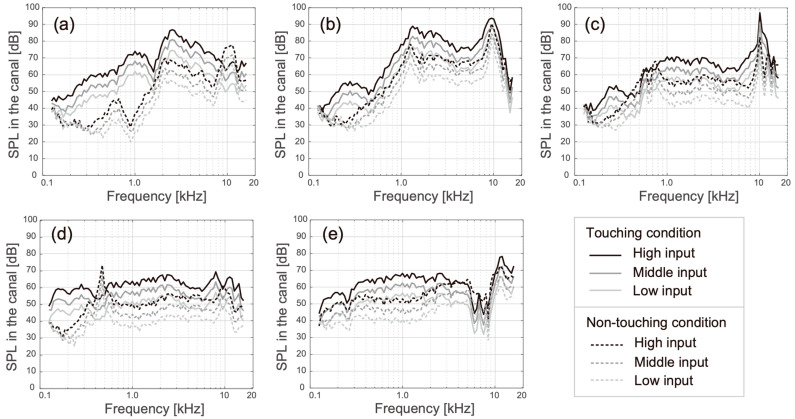
SPLs of cartilage conduction signal under the touching condition (solid lines) and airborne signal under the non-touching (dashed lines) condition. Differences in the gray scale indicate the input levels. (**a**–**e**) Represent the results for transducers A to E, respectively.

**Figure 8:**
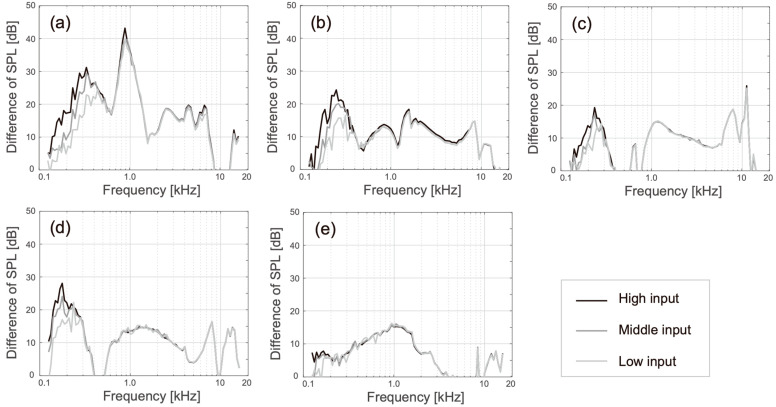
Differences between the SPLs under the touching and non-touching conditions. (**a**–**e**) Represent the results for transducers A to E, respectively.

Figure 7 shows the SPLs obtained when using the manipulated HATS under the touching (solid lines) and non-touching conditions (dash lines). The cartilage conduction signals and airborne signals were measured under the touching and non-touching conditions, respectively. The spectral shapes recorded under the non-touching condition were close to the SPLs of the airborne sounds (Figure 6). Although the SPL of transducer A decayed considerably in the low-frequency range when it was not touching the pinna simulator, this reduction was avoided by making contact with the pinna simulator (Figure 7a). The SPL difference (i.e., the SPL under the touching condition minus the SPL under the non-touching condition) was a large positive value in the frequency range below 1.5 kHz, as shown in Figure 8a. Additionally, in transducers B to E, gain in the low-frequency range could be observed; however, the amplification was not as high and it was only induced in the lower-frequency range (below 500 Hz). The amplification produced by the cartilage–air pathway could be observed in the SPL difference, as illustrated in Figure 8b–e. Among the observed results, the amplifications recorded around 250 Hz were relatively high for transducers B and D.

## 4. Discussion

Before discussing these results, we confirmed the ability of the current pinna simulator to reproduce the measured cartilage conduction sound in humans. In the previous study, the cartilage conduction sounds produced using the same hardness-adjusted HATS were compared with those produced by humans when transducer A oscillated at the canal entrance [21]. Although the corresponding values and spectral shapes were entirely similar to each other, the measured cartilage conduction sound was approximately 5 dB higher than the simulated cartilage conduction sound in the frequency range below 800 Hz. Additionally, in the frequency range higher than 5 kHz, the simulated cartilage sound was greater than the measured sound, which means that the simulated sound in this range had a lower reliability.

As shown in Figure 1, the sound transmissions during the usage of the transducers were separated into the air, cartilage–air, and cartilage–bone pathways. When a listener has normal outer ears which do not suffer from atresia of the external auditory canal, the hearing contribution via the cartilage–bone pathway is to a small extent due to the mismatch of the mechanical impedance between the aural cartilage and skull bone, and the proportion of the air and cartilage–air efforts can be quantified comparing the two cases where the transducer contacts (touching condition) or does not contact (non-touching condition) the aural cartilage [4]. In the touching condition, both the air and cartilage–air pathways work, while, in the non-touching condition, only the air pathway is functional. So, the difference between the two cases in dB indicates added sound coming through the cartilage–air pathway. For normal listeners, the transmission via this pathway is essential cartilage conduction sound.

Among the five transducers, only transducer A applied a piezoelectric driver, which was distinguished from the fitting part (the acrylic ring). Transducer A was developed for laboratory use and fitted on the averaged size of the canal opening. Transducer A could not produce airborne sound in the frequency range below 2.5 kHz (Figure 6a and dashed lines in Figure 7a); however, the cartilage conduction sound could fill this gap by contacting the pinna simulator (solid lines in Figure 7a), and the amplification by touching to the cartilage was the highest among the five transducers (Figure 8a). In the touching condition, sound arrived to the ear drum via the air and cartilage–air pathways, while, in the non-touching condition, it arrived only via the air pathway (Figure 1). Therefore, the difference between the two conditions theoretically indicates the amount of cartilage conduction sound via the cartilage–air pathway. Because the drive part of transducer A is long, thin, and located away from the entrance to the canal, the transducer can minimize the airborne sound and maximize the cartilage conduction sound. Furthermore, a larger ring-shaped fitting part extended the boundary of the canal’s entrance, which means that increased contact pressure and a larger contact surface may maximize the cartilage conduction sound. In fact, the amplitude of the sound produced by the cartilage conduction transducer increased with an increasing application force at the contact surface [4] and the aural cartilage was vibrated satisfactorily in the low-frequency range [34]. A consideration of the simulation gap described at the beginning of this section indicates that the solid lines shown in Figure 7a may shift upward in parallel by an additional 5 dB.

Generally, a versatile transducer should produce equivalent outputs in accordance with the frequency. In that sense, transducers D and E are ideal because of their flat spectral shapes for the airborne sound (Figure 6d and Figure 6e, respectively). Transducers D and E were developed for directly fitting on the canal opening and tragus without any supporting accessories, respectively. Transducers D and E are currently applied to sound collectors for conversation over a window counter and earphones for listening to music, respectively. The cartilage conduction sound amplitude was greater for transducer D than for transducer E, as shown in Figure 8d and Figure 8e, respectively. The shape of transducer D is designed to fit the canal entrance, and thus, its contact surface may be larger than that of transducer E. In contrast, transducer E was placed on the ear tragus, which was located 1 cm away from the entrance to the canal, because the tragus is one of the best positions to maximize the transducer–cartilage coupling [34,35]. The flexible wire arm was designed to hold the head softly enough to enable the use of the device over long periods (Figure 5b). Therefore, transducer E could not realize sufficient amplification via the cartilage–air pathway.

Although transducers B and C had two resonance peaks in their airborne sound characteristics (Figure 6b and Figure 6c, respectively), the lower peak around 1 kHz faded into the cartilage conduction sounds (solid lines in Figure 7b and Figure 7c, respectively). The higher peak around 10 kHz remained visible against the cartilage conduction sounds; however, our developed pinna simulator overaccentuated the simulated SPL in the frequency range above 5 kHz [21], and the resulting simulation errors may have emphasized the higher peaks. The cartilage conduction sounds were comparable with those of transducers B and C. Transducers B and C were both smaller in size than the canal entrance (Figure 3b and Figure 3c, respectively); therefore, they were simply placed on the entrance without any contact pressure. These transducers are embedded within ear plugs during actual use. Transducer B has not been installed in any product yet, while transducer C with the ear plugs is used for commercially available hearing aids. When the fitting parts are designed to maximize the contact pressure and the contact surface in a painless manner, the cartilage conduction sounds may then be greatly improved.

Which factor in the transducers influenced the different gains in cartilage conduction sound? To discuss the question, we calculate the averaged SPL of cartilage conduction sound, as shown in Figure 8. In this calculation, the negative values (e.g., around 10 kHz in Figure 8a) were excluded, and the used data were only the SPLs in response to the high input (black lines in Figure 8). The averaged SPLs of the cartilage conduction sound were 19.26 dB for transducer A, 11.61 dB for transducer B, 10.41 dB for transducer C, 12.03 dB for transducer D, and 8.35 dB for transducer E. To compare these values, we estimate the rough value of the contact surface to the entrance of the canal or ear tragus. Transducer A has a ring shape, so it can be assumed that the lower half part of the rim (exterior edge of the ring) touches the entrance of the canal. Because the thickness of the ring is 5 mm, the contact surface area can be estimated as 126 mm^2^. Transducer B may contact at the larger face of the cube shape, so the contact surface is 88 mm^2^. Similarly, transducer C contacts at the circle face of the disk, so the contact surface area is 95.03 mm^2^. Transducer D has a ball shape, so the contact surface is likely to be a lower half part of the inner hemisphere, as shown in Figure 5a. This prediction derives that the contact surface should be 95.03 mm^2^. Transducer E has a triangular-prism shape, and it contacts the ear tragus at the triangle face. When the longest side is assumed roughly as the hypothenuse of a right triangle, the contact surface area becomes 297 mm^2^.

When the generation efficiency of the cartilage conduction sound is expressed by the averaged SPL per the contact surface area, they were 0.15 dB/mm^2^ for transducer A, 0.13 dB/mm^2^ for transducer B, 0.11 dB/mm^2^ for transducer C, 0.13 dB/mm^2^ for transducer D, and 0.03 dB/mm^2^ for transducer E. The generation efficiency of transducer E was much lower than those of the other transducers. One of the reasons for this is the overrated contact surface area. The bumpy surface around the tragus may reduce the contact area in the triangle face. Although the tragus is estimated to be an appropriate position to fit the cartilage conduction transducer [34,35], the area of tragus is too small and bumpy, so the pinpoint stimulation on it is so hard. Wearing and fixing a transducer on the entrance of the canal seems to be the most reasonable way of oscillating the aural cartilage effectively. 

In transducers A to D, the maximum generation efficiency was presented by transducer A. It seems that the SPL of the cartilage conduction sound may be determined not only by the contact surface area, but also the contact pressure (application force). Transducers B to D were put on the entrance of the canal without any application force; however, transducer A pushed the boundary of the canal’s entrance softly. According to the previous study, the relationship between the SPL of the cartilage conduction sound and application force is 34 dB/N below 1 N of force [4]. If the application forces on putting transducers B and D can be assumed to be 0 N, the application force for transducer A becomes 0.08 N ($\left(19.26-0.13\times 126\right)/34$). Compared with the required application force of a bone conduction transducer (1 N), we can understand that the required force for oscillating the aural cartilage is much lower.

## 5. Conclusions

To evaluate the performances of five transducers that were developed specifically to produce cartilage conduction sounds, airborne sounds and cartilage conduction sounds were measured using a condenser microphone and a custom HATS with a manipulated ear pinna simulator, respectively. The ring-shaped transducer (transducer A) was able to minimize the airborne sound and maximize the cartilage conduction sound because the contact pressure and contact surface with the canal entrance appeared to be the largest among the five transducers. To maximize the cartilage conduction sound, it is important to design the fitting part to maximize both the contact pressure and the contact surface within the range in which the user does not feel pain. In cases where it is necessary to maintain the ventilation with respect to the external auditory canal, the ring-shaped fitting part may be the optimal choice.

## Data Availability

Not applicable.
